# Round the Hospitals

**Published:** 1918-01-26

**Authors:** 


### ROUND THE HOSPITALS.

THE new member for East Islington, Mr. E. Smallwood, devoted his maiden speech in the House of Commons on the 17th inst. to an account of how the war had smitten him through his two sons, both of whom are dead. Everybody's sympathy naturally goes out to Mr. Smallwood, whose experiences have been cruel and exceptional. His account of what happened to his eldest son we give *verbatim*: "I was wired for six months ago to go to my elder son, the only one left. I went out to the Front. He had been wounded three times. After the second time I tried to use a little influence to get him a rest at home. I could not do it. The officer himself must ask. That is not the type of young man we send to the Front; they will not ask. That is not the spirit of the men who are bred in Great Britain—not the best of them. There are many men who have gone out and got a touch of trench fever and come back and not gone out again; but they are not the type of men who have made Great Britain. He would not ask."

**ROUND THE HOSPITALS—(*continued*).**

"He was wounded a third time. I went out to see him. I was there in time. The second day passed and the third day came. I said to the doctor, 'I do not like the look of the boy.' I saw the sister. The rules of the military hospital were: Be there at two, not five minutes before; leave at five, not five minutes afterwards. I said to the sister, 'The boy is not looking what he ought to do,' and begged her to let me stop the night. She said she could not, strict orders being given that no one had to stop after five. But I said, 'I have got him to sleep; he begged me to stop.' She said, 'I am sorry I cannot do it. The Colonel has given strict orders that no one shall stop after five o'clock.' I begged that I might stay. 'No,' she said, 'the matron will be round shortly, and you must not do so, but if you like to stop on your own responsibility you must risk it.' I stayed hidden there for three hours behind a screen. Then the matron came round, angry because she found me, and insisted upon my leaving. I told her the circumstances, and I asked for the Colonel. Not one single budge would be made, not one single step would be taken. It was the last night my boy lived, and I was not permitted to stop with him. I felt—as any man who had been treated in that way would feel—I felt that the soullessness of the War Office is such as cannot be understood by the people outside."

In considering the circumstances mentioned by Mr. Smallwood we must remember that it has taken over thirty years to get our voluntary hospitals into a state of efficient administration under civil conditions, where a similar occurrence to-day would, we hope, be impossible. Had Mr. Smallwood been directed, as all friends in similar cases should be by the War Office, to go direct to the O.C. when any difficulty arises, he would have proceeded to the Commanding Officer's office when the sister took up the position described, would have laid the circumstances before him, and asked for permission to remain. Then everything would have been in order, and our careful inspection of hospital administration and the arrangements at the Front gives us confidence that the desired permission would have been obtained and the catastrophe which followed avoided. Friends of patients should be the first to be punctilious in observing the rules, though Mr. Smallwood, naturally overcome by his only surviving son's condition, consented to be hidden for three hours, a position which in ordinary circumstances no Englishman would consent to occupy. The happenings in this case have touched the hearts of all parents, and we should like to have some explanation of the matron's action, seeing that she may or may not have acted on instructions from the Commandant.

At Salterleigh, North Devon, the funeral of Miss Godiva Marian Thorold, who died on January 15, took place on the 18th instant. For thirty-five years, from 1870 to 1905, Miss Thorold was lady superintendent of the Middlesex Hospital, where a memorial service was held in the chapel on the same day. It was attended by many members of the Board of Governors, the medical staff, and by Miss Gertrude Montgomery (matron), Miss Daunt (assistant matron), Miss Alicia Lloyd Still, the secretary and assistant secretary, the sisters and nurses, the latter of whom rendered the musical portion of the service. Miss Thorold was trained at University College Hospital, and was for many years an active member of the Council of the Royal British Nurses' Association. During the thirty-five years of her matronship she exercised great influence, and devoted the whole of her energies to the best interests of Middlesex Hospital. An interesting fact in her career is that on completing her training she acted for three successive years as holiday sister-in-charge of the hospital during the matron's absence. She thus became known to the committee and staff, and when a vacancy occurred in the matronship it was offered to her and she accepted this responsible charge. Miss Thorold's motto was "Thorough," and it was her habitual practice to learn to know thoroughly both her nurses and her patients. The latter she never failed to visit individually twice a day. Her holidays were devoted to foreign travel, in the course of which she visited the Holy Land, Egypt, Greece, Constantinople, Spain, the Pyrenees, the United States, and Corsica. She was indefatigable in working for the interests of her profession, and took an active part in all movements for the benefit of nurses.

It is a curious fact that the "self-governing nurses' associations" in all the twenty-odd years of their existence have never succeeded in effecting any improvement in the economic status of nurses, or indeed in any department of the nurses' profession. An inquiry into the history of some recent co-operative movements, or of any organised trade or profession, reveals tangible results from democratic organisation. If it were not so, the principle would have been entirely discredited. That no such results mark the work of the nurses' societies shows that there must be something radically wrong either with the principle or with the societies. A trained nurse inclines to think that the recent manifesto of the Labour party might furnish a clue. That manifesto invited the co-operation of the mere brain-worker. Should this suggestion materialise, it would probably gain for Labour the adherence of thousands of men and women not hitherto recognised as workers. The mistake made by the nurses' societies lies here: they refuse to recognise those in positions of authority as *workers*, and insist on putting them in a different class. Until every member of the profession, whether matron, assistant matron, superintendent, sister, or simple nurse, is recognised as being a worker for that profession, unity is impossible, controversy must continue, and progress be hindered at every step. If every worker is to be excluded from the counsels of her profession the moment she attains a position of responsibility,

**ROUND THE HOSPITALS—(*continued*).**

the profession will sink to the level of mediocrity. A study of agricultural development shows that it is not democratisation alone that can achieve results; it must be accompanied by sincere co-operation, and the latter is more important than the former.

At the invitation of the Lady Mayoress of Manchester a meeting was held on the 22nd instant in the Mayor's Parlour to make known in Manchester the aims and objects of the Nation's Fund for Nurses. The Right Hon. the Lord Mayor was in the chair, and the speakers were the Lord Mayor and Lady Mayoress, Viscountess Cowdray, treasurer of the Nation's Fund, Mrs. Alderton, of Colchester, and Miss Sparshott, R.R.C. The Chairman of the Committee of the Manchester Royal Infirmary started the list of contributors with a gift of £100. After the meeting Lady Cowdray left for Port Sunlight to address a meeting there, called by Miss Alison Garland, on the 23rd instant, at which Lord Leverhulme presided.

We welcome the evidence, which is continuously increasing, of the administrative activity of the members of the Poor-Law Matrons' Association. Miss Barton, the president, for instance, on the 18th instant organised a meeting at the Chelsea Infirmary and arranged for Mrs. Pember Reeves, representing the Ministry of Food, to give an address on the Food Crisis. Mrs. Reeves' object was to urge the most rigid economy not only in regard to food, but to clothing, and every article which had to be made or imported. The audience seemed chiefly interested in domestic matters, and raised a number of practical questions relating to the best way to promote economy. We are glad to record that Mrs. Pember Reeves proved to be a convinced advocate of communal kitchens for all classes of the community. We have advocated the establishment of these kitchens in poorer districts for many years; they are capable of being made a great blessing and help to all workers with families, and we hope these kitchens may soon become common to all cities and smaller communities throughout the country. The representative of the Ministry of Food was quite eloquent when she described the work that was being done by its representatives in teaching cooking from a scientific point of view, with the underlying object of securing that "not even a drop of grease should be wasted." The practical success of the meeting and of Mrs. Pember Reeves was demonstrated by the number of matrons present who asked her to visit their institutions. We are asked to announce that any matron desirous of organising a meeting on these subjects should address a letter to Mrs. Pember Reeves, Ministry of Food, Grosvenor House, Upper Grosvenor Street, W., when arrangements can be promptly made.

We are glad to hear from an authoritative source that, "Miss Kathleen Stewart's resignation of the post she has so nobly filled at the York County Hospital for four years, to the apparent entire satisfaction of all in authority, and of the public," has moved Sir Frederick Milner to write to the committee expressing in strong terms the "indignation of the county at the committee's action." We are given to understand from the same authority "that the matter at issue is too trifling to be brought into account," and further that Miss Stewart is admitted to have been entirely in the right. We hope Sir Frederick Milner, with his usual public-spirited persistence, will not rest until he gets an adequate explanation and guarantees for Miss Stewart's successor. It is time that the management at the York County Hospital should be modernised, that no cliques should be associated with it, and steps taken to secure that the best interests of the patients, and the credit of the hospital in future, may be made the first considerations by the committee. The alternative is that a new committee be appointed through the action of the County Governors in the best interests of all concerned.

Miss Margaret K. Steele, on the 18th instant, was appointed Superintendent of Nurses to the York County Hospital, vice Miss Stewart, resigned. Miss Steele obtained her certificate at St. Bartholomew's Hospital, London, and has since occupied the positions of sister at the Royal Hospital for Sick Children, Edinburgh, sister housekeeper at the London Homœopathic Hospital, and for the last seven years of assistant matron of St. Bartholomew's Hospital, Rochester. Miss Steele has so had very useful experience, and we wish her all success as matron of the York County Hospital.

The statement issued by the Chairman of the Royal Commission on the Sugar Supply with regard to sugar for jam-making, has been generally accepted as fatal to the possibility of home-made jam, for the statement published was to the effect that in the coming summer no sugar, except that which could be saved from the weekly ration in each household, would be available for jam. This made it appear that hospitals and convalescent homes throughout the country, many of which, as we have shown, effected useful economies in 1917 by their skill in fruit-preserving and jam-making, would be debarred from these industries in 1918. We are glad to be able authoritatively to state that the Food Controller, in consultation with Sir Charles Bathurst, hopes to be in a position shortly to make an announcement, which will effectively save the fruit crop of 1918, and prove fair to the grower and consumer alike. The whole question of the allocation of such sugar as may be available in the hands of the Sugar Commission for jam-making is being investigated in all its bearings with this end in view. The former announcement was made with the object of advising fruit-growers, who are in a position to set aside part of their ration for the purpose of jam-making, to begin to do so at once in case other supplies should not be forthcoming later on.

